# Relative telomere length in dairy calves and dams undergoing two different methods of weaning and separation after three months of contact

**DOI:** 10.1371/journal.pone.0319156

**Published:** 2025-03-17

**Authors:** Janja Sirovnik, Rebecca Simon, Anina Vogt, Kerstin Barth, Steve Smith, Susanne Waiblinger, Gesine Lühken, Uta König von Borstel

**Affiliations:** 1 Clinical Department for Farm Animals and Food System Science, Centre for Animal Nutrition and Welfare, University of Veterinary Medicine, Vienna, Austria; 2 Animal and Pathogenetics, Department of Animal Breeding and Genetics, Justus Liebig University Giessen, Giessen, Germany; 3 Department of Animal Breeding and Genetics, Chair of Animal Husbandry, Behaviour and Welfare, Justus Liebig University Giessen, Giessen, Germany; 4 Forestry and Fisheries, Institute of Organic Farming, Johann Heinrich von Thuenen Institute, Federal Research Institute for Rural Areas, Westerau, Germany; 5 Department of Interdisciplinary Life Sciences, Konrad Lorenz Institute of Ethology, University of Veterinary Medicine, Vienna, Austria; Michigan State University, UNITED STATES OF AMERICA

## Abstract

Telomere length (i.e., the length of the repeated sequences of DNA at the end of chromosomes) is a promising indicator of overall stress. Our study aimed to compare the effects of a stress-inducing separation process between dams and their calves, with either a gradual or a nose-flap separation method after a three-months dam-calf contact since calving, on relative telomere length (RTL). Due to their nature, the nose-flap and gradual separation method have different effects on behaviour, stress hormone levels and physical development during and after dam-calf separation, which requires an overall measure of the weaning and separation stress during both procedures. We also investigated correlations between behavioural and other physiological stress indicators on RTL. We found no significant effect of the weaning and separation method on RTL in dairy calves after weaning and separation from their dams, but a tendency for shorter RTL in gradually separated dams compared to nose-flap separated dams. No correlations between behavioural and other physiological stress indicators and RTL were found, which may be due to a short interval between the two RTL measurement points. Future studies should aim to analyse the effect of various separation methods over a longer period and preferably include a non-separation group as reference.

## 1. Introduction

Telomere length is thought to be able to integrate welfare states over time and thus is considered a cumulative welfare indicator [1]. Telomeres are repetitive sequences of DNA at the ends of chromosomes that protect the cell’s genomic stability [2]. While telomere shortening occurs with every cell division [3], evidence from humans indicates that average telomere length declines at an accelerated pace due to negative life experiences in a cumulative and dose-dependent manner [1,4]. Furthermore, stress-related telomere shortening can be mitigated and possibly even reversed by positive lifestyle interventions [5]. Meta-analyses confirm telomere length to be a good indicator of psychological and physiological stress, including social stress, offspring removal and diet, across species [6,7]. In cattle, telomere length is influenced by heat stress [8], but the impact of social aspects including dam-calf separation has not yet been investigated.

Dam-calf separation is a known psychological stressor in cattle. It results in a change of behaviour indicating stress in dams and calves. After separation, effects such as increased locomotion and vocalization, reduced time performing grooming, lying down and feeding can last for days [9,10]. The separation method likely affects welfare of both dams and calves. Dams are more motivated to re-unite with suckling calves than with calves that are prevented from suckling them [11]. Thus, dams may benefit from preventing suckling before permanent physical removal of calves (i.e., permanent total separation) as reported in beef cattle [12,13]. Furthermore, as indicted by behavioural parameters, preventing suckling through either udder-nets [10] or nose-flaps [14,15] reduces post separation stress in dairy and beef calves separated from their dams or foster cows. Thus, fitting calves with nose-flaps before permanent total separation (i.e., nose-flap separation) will likely have a less negative impact on calves undergoing separation, compared to abrupt separation [14,16]. However, it is possible that the nose-flap separation distributes stress over the whole separation period as animals react to each of the two-steps in the separation process [17]. Further, nose-flaps limit physical contact between animals [18], which may lead to frustration. Additionally, just one week of nose-flap use can lead to ulcerative lesions in the nostrils and by means of dissemination of the infection to pituitary abscesses in calves [19]. Thus, even if each step of the nose-flap separation method taken individually seems to induce less stress compared to abrupt weaning and separation, cumulative stress experienced during the transition from full dam-calf contact to total permanent separation needs to be considered.

Another attempt to reduce stress during dam-calf separation is to gradually reduce the amount of the dam-calf contact prior to permanent separation (i.e., gradual separation method). Dams in semi-natural conditions spend less time with their calves with increasing calves’ age (compared to soon after birth [20]). While their dams are away, calves in semi-natural conditions spend time with other calves in creche groups supervised by a familiar cow [20]. This way the calves may already habituate to spending time separated from their dams and get accustomed to solid feed [21]. Thus, gradually reducing the time the calves are able to leave the calf area to have contact with their dams may reduce the separation stress.

Therefore, the aim of the present study was to compare the effects of gradual and nose-flap separation after a three-month period of full-time dam-calf contact, on relative telomere length (RTL) in both calves and dams as an indicator for welfare. This was part of a larger study comparing these two weaning and separation methods with behavioural and physiological indicators [22,23]. We hypothesised lower overall stress and longer RTL in calves separated with gradual separation method as the gradual nature of the separation likely allows for habituation to having less access to the dams, while the nose-flap method may introduce cumulatively more stress due to abrupt prevention of suckling and possible ulcerative injuries. In contrast, dams in the gradual separation method need to adapt to the changes of the time and duration of physical contact with their calves, which is undisturbed with the nose-flap separation method until the permanent separation. Furthermore, preventing suckling may reduce the dams’ motivation to re-unite with the calves after the permanent physical separation. Thus, we hypothesised that the nose-flap method would result in less overall stress and longer RTL in dams than the gradual separation method. We further hypothesised that RTL will negatively correlate with elevated levels of behavioural and physiological stress indicators, such as faecal cortisol metabolite levels, neutrophil: lymphocyte (N:L) ratio, vocalisations and searching behaviour. Moreover, telomere length was hypothesised to positively correlate with indicators of reduced stress (e.g., elevated levels of play behaviour in calves and rumination in cows).

## 2. Methods

### 2.1 Animals and housing

This study was part of a larger experiment, in which a number of behavioural and physiological measures were taken [22,23]. All experimental procedures were performed in accordance with the German Animal Welfare Act (Federal Republic of Germany, 2020; experiment number V244-51520/2019, MELUND Schleswig-Holstein). The experiment was carried out from November 2019 to March 2020 at the Thünen-Institute of Organic Farming in Trenthorst, Germany. Cows were kept in an open-sided barn with cubicle loose housing. They were fed a total mixed ration, composed of grass silage, corn silage, concentrate feed in the form of coarse grain and mineral feed, which was provided freshly twice a day. Cows used in the experiment were either horned or genetically hornless (polled) Black-and-White German Holstein and housed in two herds in two identical parts of the same barn with 50 cubicles each. In one herd all cows were horned, in the second herd most cows were polled, but some older cows had horns, because the herd was in a transition from formerly all-horned into a completely polled herd (Table 1).

**Table 1:** Details on the number of animals sampled. Both separation methods (nose-flap and gradual separation) were additionally both subdivided into early and late separation (with a two-week difference in the separation start). The table shows the number of individuals that were used for the analysis of telomere length.

	Nose-flap		Gradual	
	Early	Late	Early	Late
**Calves**				
**Sex**				
Males (all intact)	5	5	5	4
females	4	4	4	4
**Dams**				
**Herd**				
horned	4	5	3	5
polled	5 (1with horns)*	3 (1 with horns)*	4 (0 with horns)*	4 (2 with horns)*
**Lactation**				
1^st^ lactation	2	4	2	2
2^nd^ lactation	3	1	1	1
3^rd^ lactation	2	2	2	2
4^th^ lactation	0	0	2	1
5^th^ lactation	2	0	0	2
6^th^ lactation	0	0	0	1
7^th^ lactation	0	1	0	0

*as the polled herd was formerly also horned and at this stage in transition into a completely polled herd, there were some horned individuals remaining in the herd.

Number of cows per herd varied from 45 to 49 cows in the horned herd and 43 to 48 cows in the predominantly polled herd throughout the experiment. The herd sizes changed, when animals were removed from the herd to be either sold or moved to the calving pens or sick pens, or when primiparous cows were introduced into the herd. Cows were milked twice daily in a tandem parlour.

For each of the two herds, calves were housed in a calf area from which calves had, depending on the separation method, access to their dams in the adjacent cow barn. Calf access to the cow area was managed automatically via an automatic selection gate connected to a transponder in the neckband of the calves. When calves were restricted to the calf area, they had auditory and limited visual and physical contact with their dams through the bars in the fence separating the cow barn and the calf area (i.e., fence-line contact). The two calf areas were equipped with a resting (deep litter, 24.6 m^2^) and an activity area (rubber-coated and concrete floor, 71.9 m^2^). The groups were formed dynamically, i.e., newborn calves were continuously added to the group and older calves were taken out of the group after they had completed the weaning treatment, and group size did not exceed 23 and 20 calves for the horned and polled group, respectively. During the grazing period, calves had access to pasture together with the cow herd. Grazing period lasted until mid-November. Thus, pasture access in calves depended on the date of birth, and all but two calves received some access to pasture.

Each of the two calf areas was equipped with a concentrate feeder (Förster Technik GmbH, Engen, Germany) to which all calves had free access at all times with an allowance of 1.5 kg concentrate per animal and day distributed in portions of 50 g over the day. Individual identification of calves was ensured via a chip in the calves’ neckbands. In the calf area the calves had *ad libitum* access to water and hay. Additionally, a total mixed ration (composition was the same as for cows) was provided freshly once a day in the afternoon. No milk was provided in the calf areas.

### 2.2 Treatments

In total, 36 cows and their calves were included in the larger experiment [22,23]. The 36 cow-calf pairs were evenly distributed amongst two separation methods according to the calves’ sex (male/female) and the pair’s herd affiliation (horned/predominantly polled). However, blood samples were only collected from N =  33 cows and N =  33 calves (see Table 1 for details). The age of cows included in the present study ranged from two to nine years, with an average of four years (mean ± S.D. 4.0 ±  1.98 years) for both herds; 10 animals were primiparous cows. All calves were housed together with their dams in a calving pen for the first five days of life. Afterwards, the dams returned to their respective herd, and the calves were brought to the calf area. In the calf area, they were able to choose to either enter the cow herd and have contact with the dams or to stay in a calf area with their peers (i.e., away from their dams and the cow herd). Thus, they had full time cow-calf contact until the weaning and separation treatments started. Detailsed description of all cow-calf related terms can be found in [18].

To separate the calves from their dams we used two methods: (1) **gradual separation** (by stepwise reduction of the time of dam-calf contact) and (2) a **nose-flap separation** method (Table 2). All calves in the nose-flap separation method were fitted with a nose-flap (Quiet wean; JDA Livestock Innovations, Canada) at the beginning of the first week of the three-week separation process. Nose-flap separated calves continued to have whole-day full contact to their dams for two weeks with the nose-flap, after which the nose-flap was removed. Simultaneously with the nose-flap removal, dam-calf pairs in the nose-flap separation only had fence-line contact (i.e., calves could no longer join the cow-herd) in the third week. In the gradual separation method, calves’ access to the cow herd was reduced from whole-day contact to half day contact (i.e., 7 hour contact/day between morning and afternoon milking) in the first week and then morning contact (i.e., 3.5 hour contact/day after morning milking to midday) in the second week, and finally in the third week, analogous to the last phase in the nose-flap separation method, fence-line contact only. During access times, calves in the gradual separation method had unrestricted physical contact with their dams.

**Table 2:** Timeline of separation method application. Approximately half of the dam-calf pairs (n = 16) underwent a gradual separation (by gradually reducing the time of dam-calf contact) method, and the other half of the dam-calf pairs (n = 17) a nose-flap separation (with a nose-flap) method. The separation methods were additionally both subdivided into early and late separation (with a two-week difference in the separation start).

Calf’s age	Nose-flap		Gradual	
in weeks	Early	Late	Early	Late
≤11	Full time contact	Full time	Full time contact	Full time contact
12	Full time contact* ^S1^ with nose-flap	Full time contact	Half-day contact ^S1^ 8:30AM – 3:30 PM	Full time contact
13	Full time contact with nose-flap	Full time contact	Morning contact 8:30 AM – 12 PM	Full time contact
14	Fence-line contact	Full time contact* ^S1^ with nose-flap	Fence-line contact*	Half-day contact ^S1^ 8:30 AM – 3:30 PM
15	Total separation ^S2^	Full time contact with nose-flap	Total separation ^S2^	Morning contact 8:30 AM – 12 PM
16		Fence-line contact		Fence-line contact*
17		Total separation ^S2^		Total separation ^S2^

*calf is weaned; ^S1^ and ^S2^ indicate time points of sample collection; ^S1^:before separation, ^S2^:after separation.

Dam-calf separation of half of dam-calf pairs in both separation methods began when the calves reached 12 weeks of age (i.e., early separation) and for the other half of the dam-calf pairs separation started at 14 weeks of calves’ age (i.e., late separation). These two age groups per separation method were implemented to account for the different ages at full milk loss, which happened in the first week with nose-flap insertion in the nose-flap separation method and at the third week with fence-line contact in the gradual separation method (Table 2). Allocation to a separation time (i.e., early and late separation) was done in a balanced way for the sex of the calves, month of the study and herd of the animals.

### 2.3 Data collection

#### 2.3.1 Physiological assessment.

*Hemogram and telomere analysis:* All blood samples were taken by a qualified person and collected from the tail vein (*Vena caudalis mediana*) in cows and jugular vein (*Vena jugularis*) in calves into EDTA-coated sampling tubes. For the **hemogram,** we collected 2ml of blood from cows and calves twice per week over the whole 3-week treatment period (see 22, 23 for details). These samples were stored at 8°C until they were collected daily by a courier to be transported to a service laboratory (SYNLAB.vet GmbH, Hamburg) for haematological analysis on the same day of blood sampling. From the hemogram, the neutrophil-to-lymphocyte (N:L) ratio was calculated by dividing the numbers of neutrophils by the numbers of lymphocytes.

On the day of treatment start (day 0 for calves and day -1 for cows, S1 in Table 2) and at the last day of fence-line contact, i.e., 21 days later (day 21 for calves and day 20 for cows, S2 in Table 2) additional blood was taken for **telomere analysis**. This meant that, telomere samples were collected at 12 and 15 weeks of calves’ age in the early separation groups and at 14 and 17 weeks of calves’ age in the late separation groups (Table 2). At these two days, we collected the blood into 9 ml EDTA tubes and pipetted 2ml into the smaller EDTA tubes for the hemogram. The remaining EDTA blood was then refrigerated at -20°C within 1 h after collection. Every second week the blood was transferred to a different freezer in which samples were stored at -80 °C until the end of sample collection. Afterwards the samples were shipped to the Institute of Animal Breeding and Genetics at the Justus Liebig University Giessen for DNA extraction and further telomere analyses.Relative telomere length is expressed as the amount of telomeric DNA in relation to the amount of a reference non-variable copy number (non-VCN) gene that is constant in copy number. Relative telomere length of blood samples was estimated using the real time quantitative polymerase chain reaction (qPCR) method first established by Cawthon (2002) [24]. The reference gene *B2M* (*beta-2-microglobulin*), which is known to have a constant copy number [25], was used to quantify baseline DNA levels in samples and thus allows measurement of the amount of telomeric DNA relative to genomic DNA input (i.e., RTL). For the qPCR two pairs of previously published primers [24,26,27] were used (S1 Table in supplementary material). Primers were not used in a multiplex approach, but reactions for the reference gene and the amplifications of the telomeres were always run on the same plate for the same individual.

For inter- and intra-run calibration standards and controls, consisting of a negative control, a calibrator sample and a 1:4 serial dilution of a standard sample, were included in each 96-well qPCR plate. Samples were mixed with primer pairs (telomere or reference gene *B2M*) and the LightCycler 480 SYBR Green I Master mix (Roche Diagnostics, Germany) resulting in a total volume of 20µl per well. Controls and sample preparations were placed by hand onto the qPCR plate as triplicates, the latter one in a randomized order. However, this was done in a way that a single cow-calf pair from all four separation method and separation time combinations was analysed simultaneously on a given plate, and samples collected before and after exposure to each separation method were randomly assigned to a plate for each individual. This approach avoids potential confounds due to plate effects and run-to-run variation. Sample position remained the same before and after for most samples except for the cases when one sample had to be analysed again due to the failed quality control. This was the case for 6 cow and 8 calf samples. Those samples passed quality control at the second attempt. Run specifications were chosen according to Ilska-Warner et al. [26]. Raw data without baseline correction was extracted from the BioRad software for further analysis with LinRegPCR software [28].

*Faecal glucocorticoid (i.e., cortisol) metabolites (FGCM):* In both cows and calves, faecal samples (minimum 5g) were collected twice a week at 07:30 a.m. (with a deviation of ± 60 minutes) by rectal stimulation (calves) or taken directly from the rectum (cows) from days -1 to 20 and 0 to 21 (cows/calves respectively) relative to the start of the separation methods. These samples were homogenized and transferred into commercial faecal sampling tubes, which were promptly placed into a cooling box and then transferred to a freezer at − 20°C within 30 minutes. The samples remained stored at this temperature until further analysis.

The analysis of faecal samples in the laboratory followed a well-established protocol [29] utilizing an 11-oxoetiocholanolone enzyme immunoassay (EIA). This method has been identified as suitable for faecal cortisol metabolite (FGCM) analysis in cows [30] and calves [31].

*Average daily gain (ADG, kg/d):* Body weight of calves was determined at 7-day intervals between day 0 to 21 relative to the start of the separation methods, using a commercial cattle weighing scale (Patura Wiegekäfig, Wiegeset S1, Accuracy ±  1%). For the analysis, average daily weight gain (kg/d) for every week was calculated by dividing the weekly gain by 7 days.

*Daily milk yield:* Milk yield of the cows was recorded between days -7 and + 27 relative to treatment start twice daily during milking with the Dairy Plan C21 herd management software (GEA Farm Technologies GmbH, Bönen, Germany). Cows were milked from about 05:15-08:30 am in the morning and 3:30-6:45 pm in the evening in a 2 × 4 tandem parlour (GEA Farm Technologies GmbH, IQ cluster, 38kPa, vibration stimulation, automatic stripping device and cluster removal).

#### 2.3.2 Pathological assessment.

*Wounds of the nasal septum:* As nose-flaps can lead to wounds and infection of the nasal septum [32], calves in the nose-flap separation method were checked for purulent and/or bloody excretion at the nasal septum after the removal of the nose-flaps (i.e., two weeks after the insertion).

#### 2.3.3 Behaviour assessment.

Direct observation of calf and cow behaviour was conducted consistently by the same observer on four consecutive days per week between days − 7 to + 21 (calves) and + 27 (cows) relative to the initiation of the separation methods in all calf and cow areas. However, observations of the calves’ lying areas with the calf brush and the calves’ feeding areas were conducted via video recordings since these were not simultaneously visible with the rest of the barn during direct observations. The following behaviours were observed through direct observation:

1. **Vocalization**: Frequency was recorded continuously for both calves and cows. Vocalization was defined as clearly audible sounds produced through the mouth of a calf or cow [14].
2. **Searching behaviour**: Occurrence was recorded at intervals of every 3 minutes for both calves and cows. Searching behaviour was defined as movements parallel to, within 1 meter of, the pen partition, standing with its head through the selection gate, or standing beside the pen partition (within a radius of 2.5 meters) with the head elevated and eyes and ears focused in the direction of the calf’s or cow’s section, including scanning (adapted from [14,17]).
3. **Locomotor play**: Duration was recorded continuously for calves. Locomotor play was defined as galloping, jumping, leaping, bucking, or buck-kicking of a calf (after [33]).
4. **Social play**: Duration was recorded continuously for calves. Social play was defined as two calves standing front to front, pushing, rubbing, or butting head against head/neck without force, often including rotating head movements (adapted from [33]).
5. **Object play**: Duration was recorded continuously for calves. Object play by the calf was defined as butting water bowl, hayrack, or bars in the pen, standing up or butting straw, or rubbing head, throat, or neck in straw, kneeling down on the two forelegs [33].

Locomotor, social, and object play behaviours were combined into ‘**total play behaviour**’ for analysis.

Direct observations occurred 2 hours following morning milking, 1 hour before the start of evening milking, and 2 hours following evening milking. These time points were selected because cows and calves were accustomed to being separated and reunited around milking times, exhibiting the most activity during these periods. Observations following the termination of morning and evening milkings began as soon as the cows and calves were allowed to reunite. The observer alternated between the two herds every 30 minutes, ensuring that each cow-calf pair was observed for a total of 2.5 hours daily. The start of observations alternated between the horned herd and the polled herd, with the schedule alternating between even and odd calendar weeks.

Video analysis of **total mixed ration (TMR) feeding time** and **brush use** in calves was conducted on the same four consecutive days as direct observations. Jovision Infrared Network Cameras with Jovision NVR System Software (Version 2.0.1.49) were utilized for this purpose. Total mixed ration feeding time was defined as the duration during which the calf’s head was placed through the feeding rack, with the ears positioned in front of the rack. Brush use was defined as any physical contact with the brush by any body part of the calf while being automatically brushed or while scratching itself against the brush. This definition was adapted from [34].

Continuous observation was employed for both total mixed ration feeding time and brush use analyses. Animal behaviour was continuously recorded in intervals of 6 hours: night (00:00–06:00), morning (06:00–12:00), afternoon (12:00–18:00), and evening (18:00–00:00). Specific intervals on days of treatment change were subsequently removed from the dataset; for example, the night interval of the day of nose-flap insertion, as the nose-flap was inserted in the morning. Analysis was conducted by four different observers for TMR feeding time (with Cohen’s kappa ≥  0.91) and three different observers for brush use (with Cohen’s kappa ≥  0.88). One observer had to be excluded due to insufficient inter-observer reliability for brush use. The software BORIS ([35], Version 7.9.22) was employed for the analysis of the video recordings. Cameras automatically switched to infrared mode in dark light conditions. For calf identification during the infrared mode, photos of the calves’ individual coat patterns were utilized.

Automatic assessment was utilized to analyse the lying behaviour and locomotor play of calves. To assess **lying behaviour**, accelerometers (Hobo Pendant G data loggers, Onset Computer Corporation) were attached to 28 out of the 36 calves (14 per treatment). The 3-axis accelerometers were positioned directly above the metacarpal joint on the medial side of the left hind leg, tilted by 90° to ensure a better fit to the calves’ legs. This orientation aligned the y-axis parallel to the ground and the x-axis perpendicular to the ground, pointing downward. Data loggers recorded g-force and the degree of tilt of the x-axis at 60-second intervals from day –28 to + 21 relative to the start of the separation methods. The period from days − 28 to -22 was designated as a habituation period. Due to limited storage capacity, the accelerometers were replaced every 14 days. Data retrieval was performed using HOBOware software (Onset Computer Corporation, Version 3.7.17).

Recordings obtained during the fitting or replacement of loggers, occurring between 07:00 and 10:00 a.m. on respective days, were excluded from analysis due to their potential to disrupt the data. The degree of vertical tilt (x-axis) was then utilized to determine the lying position of the calf. Readings ≤  120° indicated that the calf was lying down, while readings >  120° indicated that the calf was standing up (adapted from [36] for the tilted logger position). Subsequently, the recorded data were refined using an event filter. This filter identified and adjusted single outlier readings. For example, if a single lying event was surrounded by standing events, or vice versa, it was adjusted to reflect the behaviour preceding it. These adjustments were made because such readings were considered potentially erroneous [37]. From this processed data, the **total lying time** as a percentage per day and the number of **lying bouts** per day were computed.

For the automatic assessment of **locomotor play**, a subgroup of calves was chosen from the original cohort. Specifically, 28 out of the 36 calves, comprising 13 in the NF treatment and 15 in the GR treatment (all but 4 calves were identical to those used for the assessment of lying behaviour), were equipped with a second accelerometer. This accelerometer was positioned on the medial side of the right hind leg and was worn continuously for 9 hours each day, from 08:00 a.m. to 05:00 p.m., on days − 4, + 3, + 10, and + 17 relative to the start of the separation methods.

The accelerometers were tilted by 90° to guarantee more precise measurements. The loggers were programmed to measure the g-force of the vertical axis (x-axis) at a rate of 1 Hz, a methodology validated by Luu et al. (2013) [38]. Subsequently, the recorded data were exported using HOBOware and analysed utilizing the peak acceleration method, as outlined by Größbacher et al. (2020) [39]. This method entails counting acceleration peaks recorded by the data loggers to identify instances of play events by the calves and thresholds to define peak accelerations were validated via video comparisons [23].

In cows, **lying behaviour** was recorded with Ice Tag leg sensors (Ice Robotics Ltd, Edinburgh, UK), which were attached to the left hind leg of each cow from days -14 to + 27 relative to treatment start. If a lying bout was < 4 minutes, it was deleted from the dataset as erroneous reading. **Rumination time** of cows was measured with RumiWatch noseband pressure sensors (Itin + Hoch GmbH, Liestal, Switzerland) from day -14 to + 27 relative to separation start. Days when leg and noseband sensors were fixed or removed (day -14 and + 27) were omitted from the data since data were only available for part of these days. Also, days -14 till -8 were omitted from the data set for rumination and lying times as habituation period.

In the final step, all data were corrected for illegitimate contact between cow and calf and ill health of the animals, as well as dams being in oestrus, before conduction of statistical analysis (see 22 and 23 for details).

### 2.4 Statistical analysis

For statistical analysis, we used the statistical software R studio [40] in R statistical program [41]. Sample size was determined a priori using G * Power 3.1, assuming a large effect (f = 0.5) and accepting an α-error of 5% and a β-error of 10%.

#### 2.4.1 Separation method.

We analysed the treatment-related data via ANCOVA, with RTL after separation (final RTL) as the outcome variable, which is a suggested method for analysing RTL data obtained by the qPCR method [42]. Predictor variables included in the global statistical model for calves were separation method (nose-flap or gradual), separation time (early or late) and their interaction, herd (horned or polled), sex, and q-PCR plate number. Predictor variables in the global model for dams were separation method, separation time and their interaction, herd, age, and q-PCR plate number. Parity was not included in the model, because previous research did not show an effect on behavioural indicators in response to separation [43] and due to a correlation between age and parity. There are individual differences in telomere length already at birth and they remain present throughout life. Thus, initial RTL was included as a covariate to correct for the ‘regression to the mean’ [44] in both dams and calves.

We selected best models using AICc and the function dredge (package *MuMIn*), and separation method and initial RTL (i.e., covariate) were fixed terms. The selected models contained separation method, separation time as predictor variables, and initial RTL as covariate. We also checked whether the presence of the purulent and/or bloody nasal septum excretion resulted in shorter RTL. For this we fitted an ANCOVA model for the calves of the nose-flap separation method using status of the nasal septum (binary response: purulent and/or bloody excretion *vs.* no or transparent excretion) and the covariate initial RTL as predictor variables.

Prior to fitting the models, we inspected the distribution of final RTL, which appeared to be symmetrically distributed. To verify the assumption of the models, residuals were checked for normal distribution using a Shapiro–Wilk test, histogram and qq-plot and for homogeneity of variance using a Levene test and by plotting residuals against fitted values. Also collinearity was not an issue (maximum Variance Inflation Factor: 1.144 [45]). To ensure that initial RTL did not differ between separation methods and separation times [46] we tested for ANCOVA’s assumption of independence of the covariate and fixed factors. This was done with an ANOVA, in which RTL before separation introduction (initial RTL) was treated as independent variable and separation method and separation time as predictor variables. We also visually inspected that there was no correlation between initial RTL and separation method and separation time with boxplots where separation method and separation time were depicted on the x-axis and initial RTL on the y-axis.

To test the significance of the predictors as a whole and avoid ‘cryptic multiple testing’ [47] we compared the fit of the full model with that of the null model comprising only the initial RTL. The estimates of the coefficients were obtained using a ‘summary.lm(model)’ function. Estimated marginal means (adjusted for initial RTL) and standard errors of final RTL from ANCOVA were calculated using the package *emmeans*.

#### 2.4.2 Behavioural and physiological stress indicators.

We separated the data for cows and calves, as well as for each separation method, calculated average values for each measure over a three-week separation period and utilized histograms to inspect the data for normality. Subsequently, we calculated correlations between final telomere length and averages of other physiological and behavioural stress indicators, as well as average daily weight gain and daily milk yield. For this correlation analysis, we used the pcor.test function with the Spearman method [48]. Additionally, we included initial telomere length as a covariate to correct for the ‘regression to the mean’. This approach helps to account for any baseline differences and strengthens the accuracy of the correlation analysis. Due to multiple comparisons we used p.adjust function with a holm method to adjust p-values.

## 3. Results

### 3.1 Separation method

The estimated marginal means and standard errors of final RTL (adjusted for initial RTL) of cows and calves in different separation methods and separation times are shown in Table 3 and model outputs are reported in S2 and S3 Tables in the supplementary material.

**Table 3:** From ANCOVA estimated marginal means (adjusted for initial RTL) and standard errors (SE) of final RTL (TL/non-NVC).

	Cows				Calves			
	n	mean	SE	P	n	mean	SE	P
**Separation method**				0.095				0.759
Nose-flap separation	17	1.36	0.08		18	1.15	0.07	
Gradual separation	16	1.16	0.08		17	1.11	0.08	
**Separation time**				0.135				0.079
Early	16	1.17	0.08		18	1.22	0.07	
Late	17	1.35	0.08		17	1.03	0.08	
**Nasal septum condition**								0.409
Excretion*					5	1.07	0.08	
No excretion*					13	1.20	0.13	

*Excretion: bloody or purulent excretion at the nasal septum where the nose-flap had contact.

In dams, the gradual separation method tended to result in shorter final RTL (1.16 ± 0.08) compared to the nose-flap separation (1.36 ± 0.08, T =  -1.725, P =  0.095), and separation time did not affect final RTL (T =  1.536, P =  0.135). In calves, separation method did not affect final RTL (Table 3 and SM 2; T =  -0.310, P =  0.759), but there was a tendency for late separation to result in shorter final RTL (1.03 ± 0.08) than early separation (1.22 ± 0.07, T =  -1.819, P =  0.079). In calves, the covariate (initial RTL) had a significant effect on final RTL (T =  2.667, P =  0.012) indicating that the initial RTL had a positive relationship with the final RTL and that it adjusted the model output of the dependent variable (final RTL). There was no effect of initial RTL (T =  1.071, P =  0.293) on final RTL in dams. Initial RTL was not different for both separation methods and separation times in either dams or calves (S5 and S6 Tables).

Six out of eighteen calves in the nose-flap separation method developed wounds with purulent and/or bloody excretion of the nasal septum. Numerically, we observed that calves with purulent and/or bloody wounds had shorter final RTL. However, testing for statistical significance revealed no effect of nasal septum wounds on final RTL (T =  -0.850, P =  0.409; S4 Table in the supplementary material). Also in this model, the covariate (initial RTL) had a positive relationship with the final RTL (T =  4.152, P =  0.001).

### 3.2 Behavioural and physiological stress indicators

None of the other physiological or behavioural measures showed statistically significant partial correlations with the final relative telomere length (RTL), as indicated in Table 4. Additionally, daily milk yield in cows (nose-flap: r_s_ =  -0.016, P =  10.953; gradual: r_s_ =  0.341, P =  0.213) and average daily weight gain in calves (nose-flap: r_s_ =  0.295, P =  0.250; gradual: r_s_ =  0.122, P =  0.653) were also not correlated with the final RTL.

**Table 4:** Partial correlation estimates (r_s_) and P-values between final RTL and the stress measures from the Spearman correlation tests.

Parameter	Cows				Calves			
	*Nose-flap*		*Gradual*		*Nose-flap*		*Gradual*	
	r_s_	P	r_s_	P	r_s_	P	r_s_	P
**Physiological stress indicators**								
N:L ratio	0.207	0.918	0.207	0.459	-0.133	1	0.228	0.79
FGCM^1^	-0.038	1	-0.039	1	-0.158	1	-0.331	0.422
**Behavioural stress indicators**								
Vocalisations	-0.187	1	-0.033	1	0.147	1	-0.1	0.714
Searching	-0.172	1	0.011	1	-0.018	1	-0.304	1
Total play^2^					-0.424	0.63	0.069	1
Locomotor play^3^					0.079	1	-0.243	1
Lying duration	-0.295	1	-0.458	0.915	-0.434	1	0.245	1
Lying bouts	0.255	1	-0.003	1	-0.258	1	0.331	1
Brush use					-0.050	1	-0.157	1
Rumination	-0.554	0.48	-0.315	1				

^1^Faecal glucocorticoid (i.e., cortisol) metabolites,

^2^direct observation,

^3^automatic assessment.

## 4. Discussion

This study compared the effects on telomere length of dams and their calves undergoing either a nose-flap or gradual separation method. Although in cattle telomere length has been widely studied mainly in relation to fitness traits associated with survival, longevity, health, and reproductive use [25,26,49], knowledge about the effect of psychological stress on telomere length is lacking in this species. This study is the first step to filling this information gap as dam-calf separation is a known psychological stressor in cattle [9,10]. We hypothesised that compared to the nose-flap separation, gradual separation would result in less overall stress and longer RTL in calves and to be cumulatively more stressful and lead to shorter RTL in dams. Indeed, our study found a tendency for shorter final RTL, indicating higher stress levels, in dams undergoing gradual separation as compared to the nose-flap separation method. However, we identified no significant effect of the separation method on final RTL in calves. We also hypothesised that behavioural and physiological indicators of stress will correlate with telomere length. This hypothesis was not confirmed as none of the measures correlated with the final RTL.

We collected samples for telomere length measurements three weeks apart – immediately before the separation process began and at the end of the fence-line contact period. Studies on other (shorter lived) species showed an effect of stress on telomere length after nine days in young and four weeks in adult animals [1]. Thus, in dams three weeks may have been a too short interval between samplings. Additionally, behaviour analysis of the same animals revealed that differences between separation methods in the number of vocalisations and proportion of searching behaviour in cows and calves were greater during the fence-line contact phase compared to other separation phases (i.e., insertion of nose-flaps and reducing the contact time) [22,23]. Fence-line contact was only introduced one week before the second telomere sample collection. Thus, differences in cumulative stress indicators such as RTL between separation methods as well as correlations between behavioural and physiological stress indicators and RTL would likely be greater at a later sampling time point, i.e., at least nine days and four weeks after introduction of fence-line contact in calves and dams, respectively.

Given that our sampling interval was short, RTL results probably reflect mostly the initial phase of the separation process. The found tendency for shorter final RTL in dams of the gradual separation method compared to nose-flap separated dams, might therefore indicate that the restriction of nursing by the calves was less stressful to the dams than separation from the calf over night and part of the day. This could be explained by the fact that nose-flap calves were still present in the cow area and dams could express other types of affiliative behaviours with their calves. In this regard, it has been previously shown that suckling increases oxytocin levels [50], which is among others involved in the establishment and maintance of social bonds [51]. Likely related to this, motivation in dams to re-unite with their calves is increased in dams that are suckled by their calves [11]. This could further explain why nose-flap cows which were not suckled but had access to their calves, were less stressed than gradual reduction cows which were still suckled but were temporarily separated from their calves at the measured time point.

Additional to the suckling, various other types of interaction, which could also influence social bonding between dams and their calves, were restricted by the separation methods. For example, play and lying close to the calf were restricted in the gradually separated dam-calf pairs to the time when calves were permitted to visit the dams and nose-flap separation calves were prevented from licking the dams. However, the effects of these types of interactions on separation stress have not been studied so far.

Dams respond to vocalisations of their calves by walking towards the location of vocalisation [52]. Gradually separated calves vocalised more over the whole separation period, but especially during the last phase of separation (i.e., fence-line contact) [23]. This corresponds with the proportion of searching behaviour, which showed a similar pattern as vocalisations [23]. Thus, it is possible that dams in the gradual separation method experienced stress in response to vocalisations of their calves, especially since they could not reach the calf. The effect was minor, but our results on RTL indicate that preventing calves to lick and suckle the dams in the nose-flap separation method is overall less stressful to the dams than restricting the amount of time they could be in full physical contact with their calves in the gradual separation method.

Milk production is thought to be a physiological stressor [53]. Dams in both separation methods continued to be milked twice a day as before the separation began. Analysis of the milk yield from the dams of the same experiment showed that the nose-flap separation resulted in a higher milk yield during machine milking than gradual separation when considered over the whole 3-week separation period [22]. However, in cow-calf contact systems, milk obtained in the milking parlour is not a good indicator of milk production as it is affected by various factors related to suckling and machine milking prior to weaning, including milk ejection efficiency [54]. Furthermore, milk production has not been shown to affect telomere shortening in cattle [8]. Thus, the difference between the separation methods regarding milk production is an unlikely explanation for this trend in the effect of separation method on RTL.

There was no significant effect of separation method on RTL in calves, which is in accordance with the physiological measures from our animals as reported elsewhere: faecal cortisol metabolite concentrations and differential blood count over the whole separation process did not differ between the separation methods [23]. Behavioural indicators and some physiological measures collected for the same animals showed differing effects of the separation method on animal welfare [23] and do not offer an explanation for our findings. Although both separation methods induced behavioral and physiological distress responses, a gradual reduction of contact time to the dam was favorable compared with weaning with a nose-flap in most indicators [23]. For example, gradually weaned calves showed higher weight gains and a lower decrease in lying bouts and locomotor play levels compared with baseline, indicative of higher energy levels and a potentially less compromised welfare [23]. Moreover, presence of the nasal septum wounds was observed in reaction to nose-flap weaning and not to gradual weaning (23). As telomere shortening decellerates in response to increased locomotor levels and accelerates in wounded animals [1], it was contra to our expectations that this was not reflected in a shorter RTL in our study. However, the mechanisms of telomere shortening are complex and also may be influenced by other factors such as body weight, as in theory faster body growth requires more cell divisions and, therefore, bigger sized individuals of the same species have shorter telomeres [55].

The two separation methods likely led to different behavioural needs being unfulfilled. Nose-flaps in the nose-flap separated calves prevented suckling, and possibly restricted the ability to perform social or self-grooming [18], which could negatively affect calves’ welfare. Gradual separation limited the time allowed to spend in physical contact with the dams and other cows [18]. With advancing age, calves in semi-natural conditions spend increasingly less time with their dams and more time with other calves [56,57]. However, they stay under supervision of another cow from the herd while the dams are away [56,57]. Being forced to spend time away from any adult cow while not in contact with the dams may thus additionally negatively affect their welfare. Hence, it is likely that calves in both types of separation methods were frustrated due to unsatisfied behavioural needs, which resulted in no significant differences with regard to cumulative stress level (i.e., final RTL). In further studies, it would be interesting to investigate, if allowance of contact to one or more unrelated, adult cows could help to alleviate negative welfare effects of separation from the dam as in horses [58].

We found a tendency for shorter final RTL in calves undergoing late separation compared to early separation, whereas in dams, final RTL was not affected by separation time (early vs. late). Telomeres shorten with each cell division [59] resulting in shorter telomeres in older animals compared to younger individuals [49]. Nevertheless, there was no difference in the initial RTL suggesting that the difference in the final RTL likely originates from the separation stress. This is interesting as other data in the same calves showed that late separated calves likely experienced less stress than early separated calves as seen by a lower decrease in play behaviour compared to before separation [31]. Also non-related studies showed less separation stress in older calves as seen by fewer vocalisations and lower increase in cortisol levels after the separation [60,61]. Thus, the tendency for shorter RTL in calves of the late separation treatment in our study may point at confounding factors related to age such as increasing size and thus space requirements which may be fulfilled decreasingly in the calves’ area but are not related to separation and weaning stress itself [62,63]. However, while telomere length generally decreases with age, the rate of telomere shortening can vary [49]. This variation may account for the differences observed between the early and late separation methods.

Diet can affect telomere length in humans [64], but this effect has not been confirmed in adult cattle [8]. No information on calves could be found. Under semi-natural conditions, dams usually nurse their calves for 8–12 months, until shortly before their next calving [65]. Calves in our study were weaned at a much younger age than under semi-natural conditions. Thus, a difference of two weeks as in our study is likely too small to lead to strong differences in calves’ welfare due to changes in diet that would be reflected by RTL. Calves were also weaned at an older age compared to most other commercially kept calves, which are usually weaned at 8-12 weeks, but sometimes even as early as 6 weeks of age [66–68]. Future studies should investigate the effects of other weaning ages on RTL.

Dam-calf pairs can be separated after various lengths of contact times and with different methods. We only compared two separation methods. To get more comprehensive information about the effect of a given separation method as well as age at separation on welfare, the separation methods used in this study should be compared to abrupt separation after prolonged dam-calf contact, separation at birth as usually done in conventional dairy farms (negative control) and not separated dam-calf pairs (positive control). Furthermore, additional external factors such as weather conditions and health that were not considered in this study might have confounded the results [1,8]. This study revealed no age or sex effects on telomere length in dams and calves, respectively. Numerically it appears that cows had longer telomeres than calves, however, this was not statistically analysed and due to a high inter-individual variation in telomere length, may not be statistically significant. Telomere shortening slows-down in adult cattle and may be difficult to detect as previous research indicated [49]. Males usually have shorter telomeres compared to females [69], however it seems that the sex effect on telomere length appears later in life [70,71].

Nose-flaps caused wounds with purulent and/or bloody nasal excretion in six calves (23). Inflammations and infections may affect RTL in certain conditions through increased cell division, direct damage to telomeres via oxidative stress, or reduction of telomere repair associated with physical and psychological stress [1]. However, we did not see an effect of nasal septum wounds on final RTL. This may be due to a small number of affected calves. Additionally, it is likely that the stress associated with impaired health was not strong enough to affect RTL within the short period in our study. However, another study showed no effect of udder health and lameness in cows on RTL [8] indicating that RTL may not be a valid indicator of health in cattle.

Quantitative PCR is a standard method to measure telomere length. However, results from southern blotting, the much more laborious gold standard method, have a higher statistical power, e.g., due to lower measurement errors [42]. We cannot exclude that measurement of telomere length by southern blotting and/or in a higher number of animals would have led to higher precision. However, in this study, resources were not available to establish southern blotting. Including a higher number of experimental animals to increase the statistical power was not possible, because this study was part of a larger project on behaviour and physiology, and sample size was planned for these parameters [22,23].

## 5. Conclusion

Our study shows no significant effect of weaning and separation method on RTL in dairy calves after weaning and separation from their dams, which is in line with physiological stress indicators in other analyses [23]. However, a tendency for shorter RTL in gradually separated dams compared to nose-flap separated dams could indicate that gradual separation may be overall more stressful for dams. In contrast to previous reports [60,61], our results (i.e., shorter RTL in late separated calves) point to greater overall stress in late separated calves, though the finding was only a tendency and may be confounded with other age-related factors such as calves space requirements [62,63], which should be investigated further. Although telomeres are thought to be a promising indicator of cumulative stress [1], we suggest more precise measurement methods, e.g., southern blotting to be used in future studies on dam-calf separation stress. Further, we suggest that future studies should aim to analyse the effect of various separation methods over a longer period than 21 days, as used in this study, and ideally include a non-separation group as reference.

## Supporting information

S1 Table Primer pairs used to amplify the reference gene and the target (telomere). (DOCX)

S2 Table Model output for dams. (DOCX)

S3 Table Model output for calves. (DOCX)

S4 Table Model output for nose purulent and/or bloody excretion in calves in the nose-flap separation method. (DOCX)

S5 Table Model output of the independence testing in dams. (DOCX)

S6 Table Model output of the independence testing in calves. (DOCX)

S1 Data Data used for the analysis of calf telomere length in relation to behavioural and physiological outcomes: calf.correlations.csv. (CSV)

S2 Data Data used for the analysis of dam telomere length in relation to behavioural and physiological outcomes: cow.correlations.xlsx. (XLSX)

S3 Data Data used for the analysis of telomere length in relation to treatments: combined_na_removed.xlsx. (XLSX)
