# Supplementary material for: Relative telomere length in dairy calves and dams undergoing two different methods of weaning and separation after three months of contact
Source: PLoS One. 2025 Mar 17;20(3):e0319156. doi: 10.1371/journal.pone.0319156 (PMC11913301; doi:10.1371/journal.pone.0319156)
Supplement: S1 Table — (DOCX) [file pone.0319156.s001.docx]

Table SM 1. Primer pairs used to amplify the reference gene and the target (telomere).

| **Primer name (concentration)** | **Primer sequence** | **Reference** |
| --- | --- | --- |
| Telomer 1b (10 pmol)  Telomer 2b (10 pmol) | 5’-CGG TTT GTT TGG GTT TGG GTT TGG GTT TGG GTT TGG GTT-3’  5’-GGC TTG CCT TAC CCT TAC CCT TAC CCT TAC CCT TAC CCT-3’ | (27,28) |
| B2M 1b (10 pmol)  B2M 2b (10 pmol) | 5’-GCTTCTGACACAACTGTGTTCACTAGC-3‘  5‘-CACCAACTTCATCCACGTTCACC-3‘ | (25,27) |
