# Supplementary material for: Relative telomere length in dairy calves and dams undergoing two different methods of weaning and separation after three months of contact
Source: PLoS One. 2025 Mar 17;20(3):e0319156. doi: 10.1371/journal.pone.0319156 (PMC11913301; doi:10.1371/journal.pone.0319156)
Supplement: S2 Table — (DOCX) [file pone.0319156.s002.docx]

Table SM 2. Model output for dams.

| Response: final RTL | Estimate | SE | T value | P-value |
| --- | --- | --- | --- | --- |
| Intercept | 0.944 | 0.296 | 3.191 | 0.003 |
| S. method_gradual | -0.199 | 0.115 | -1.725 | 0.095 |
| S. time_late | 0.181 | 0.118 | 1.536 | 0.135 |
| Initial RTL | 0.269 | 0.251 | 1.071 | 0.293 |
