# Supplementary material for: Relative telomere length in dairy calves and dams undergoing two different methods of weaning and separation after three months of contact
Source: PLoS One. 2025 Mar 17;20(3):e0319156. doi: 10.1371/journal.pone.0319156 (PMC11913301; doi:10.1371/journal.pone.0319156)
Supplement: S3 Table — (DOCX) [file pone.0319156.s003.docx]

Table SM 3. Model output for calves.

| Response: final RTL | Estimate | SE | T value | P-value |
| --- | --- | --- | --- | --- |
| Intercept | 0.879 | 0.171 | 5.139 | 0.000 |
| S. method_gradual | -0.033 | 0.106 | -0.310 | 0.759 |
| S. time_late | -0.191 | 0.105 | -1.819 | 0.079 |
| Initial RTL | 0.288 | 0.108 | 2.667 | 0.012 |
