# Supplementary material for: Relative telomere length in dairy calves and dams undergoing two different methods of weaning and separation after three months of contact
Source: PLoS One. 2025 Mar 17;20(3):e0319156. doi: 10.1371/journal.pone.0319156 (PMC11913301; doi:10.1371/journal.pone.0319156)
Supplement: S4 Table — (DOCX) [file pone.0319156.s004.docx]

Table SM 4. Model output for nose purulent and/or bloody excretion in calves in the nose-flap separation method.

| Response: final RTL | Estimate | SE | T value | P-value |
| --- | --- | --- | --- | --- |
| Intercept | -0.173 | 0.337 | -0.514 | 0.615 |
| Excretion_yes | -0.135 | 0.159 | -0.850 | 0.409 |
| Initial RTL | 1.033 | 0.249 | 4.152 | 0.001 |
