# Supplementary material for: Relative telomere length in dairy calves and dams undergoing two different methods of weaning and separation after three months of contact
Source: PLoS One. 2025 Mar 17;20(3):e0319156. doi: 10.1371/journal.pone.0319156 (PMC11913301; doi:10.1371/journal.pone.0319156)
Supplement: S5 Table — (DOCX) [file pone.0319156.s005.docx]

Table SM 5. Model output of the independence testing in dams.

| Response: initial RTL | Estimate | SE | T value | P-value |
| --- | --- | --- | --- | --- |
| Intercept | 1.115 | 0.069 | 16.234 | 0.000 |
| S. method_gradual | 0.097 | 0.082 | 1.183 | 0.246 |
| S. time_late | 0.131 | 0.082 | 1.596 | 0.121 |
