# Supplementary material for: Relative telomere length in dairy calves and dams undergoing two different methods of weaning and separation after three months of contact
Source: PLoS One. 2025 Mar 17;20(3):e0319156. doi: 10.1371/journal.pone.0319156 (PMC11913301; doi:10.1371/journal.pone.0319156)
Supplement: S6 Table — (DOCX) [file pone.0319156.s006.docx]

Table SM 6. Model output of the independence testing in calves.

| Response: initial RTL | Estimate | SE | T value | P-value |
| --- | --- | --- | --- | --- |
| Intercept | 1.348 | 0.147 | 9.172 | 0.000 |
| S. method_gradual | -0.158 | 0.171 | -0.924 | 0.362 |
| S. time_late | -0.029 | 0.171 | -0.172 | 0.865 |
